# One Health Investigation of Stage-Dependent Antimicrobial Resistance Patterns Across Intermediate and Ripened Dairy Matrices: The Tyrovolia–Kopanisti Paradigm

**DOI:** 10.3390/microorganisms14030712

**Published:** 2026-03-22

**Authors:** Georgios Rozos, Konstantina Fotou, Vaia Gerokomou, Konstantina Nikolaou, Aikaterini Dadamogia, Lampros Hatzizisis, Ioannis Skoufos, Athina Tzora, Eugenia Bezirtzoglou, Chrysoula (Chrysa) Voidarou

**Affiliations:** 1 Laboratory of Animal Health, Food Hygiene and Quality, Department of Agriculture, School of Agriculture, University of Ioannina, 47150 Arta, Greece; kfotou@uoi.gr (K.F.); v.gerokomou@uoi.gr (V.G.); knikolaou@uoi.gr (K.N.); kdadamogia@gmail.com (A.D.); tzora@uoi.gr (A.T.); 2 Laboratory of Animal Science, Nutrition and Biotechnology, Department of Agriculture, School of Agriculture, University of Ioannina, 47150 Arta, Greece; lamprosxatz@uoi.gr (L.H.); jskoufos@uoi.gr (I.S.); 3 Laboratory of Hygiene and Environmental Protection, Department of Medicine, Democritus University of Thrace, Dragana, 68100 Alexandroupolis, Greece; empezirt@yahoo.gr

**Keywords:** *Lactobacillus*, AMR, “Tyrovolia”, “Kopanisti”, *tet*M, *tet*K, *erm*B, *bla*TEM, *cat*

## Abstract

Antimicrobial resistance (AMR) emerges and circulates across interconnected human, animal, food, and environmental reservoirs; however, food fermentation systems remain underexplored as indicators of local AMR pressure, even though artisanal dairy fermentations may function as natural sentinels of AMR. In this study, we used an artisanal dairy fermentation chain as a One Health model to investigate whether environmentally exposed lactobacilli can reflect stage-associated shifts in resistance. A total of 1.085 isolates representing 16 *Lactobacillus* species were recovered from the same artisanal dairy matrix at two fermentation stages: day 5 (“Tyrovolia”; n = 518) and day 30 (“Kopanisti”; n = 567). Susceptibility to 14 antibiotics was evaluated by broth micro-dilution, and *L. acidophilus* was further screened for selected resistance genes. Overall resistance increased significantly from 69.88% (362/518) at day 5 to 77.25% (438/567) at day 30 (*p* = 0.0059), while multidrug resistance rose from 37.57% to 60.73% of resistant isolates (*p* < 0.001). Across the 224 species–antibiotic combinations examined, 129 (57.58%) showed an increased upper MIC limit at day 30, and resistance increased significantly for 9 of the 14 antibiotics tested, with the largest rises observed for metronidazole (RR = 7.67), chloramphenicol (RR = 5.74), quinupristin/dalfopristin (RR = 4.11), vancomycin (RR = 2.78), and trimethoprim (RR = 2.43). In contrast, erythromycin and oxytetracycline resistance declined significantly at the ripened stage. In *L. acidophilus*, 21 resistance genes were detected in 14/70 day-5 isolates and 19 genes in 13/71 day-30 isolates, but marked genotype–phenotype discordance was observed, including matrix-dependent expression patterns for *tet*M, *erm*B, and *bla*TEM. Collectively, these findings show that environmentally exposed artisanal dairy fermentations can enrich resistance phenotypes and may serve as sensitive sentinels of AMR dynamics at the human–animal–environment interface.

## 1. Introduction

Antimicrobial resistance (AMR) is widely recognized as one of the major global health challenges of the Anthropocene, undermining the prevention and treatment of bacterial infections in humans and animals and threatening the safety and sustainability of food systems. Resistance has increased worldwide, particularly among clinically important bacterial pathogens, leading to rising morbidity, mortality, and economic burden. Current projections suggest that, without effective mitigation, the global annual economic losses attributable to AMR may increase substantially by 2050, from hundreds of billions of USD today to over one trillion USD [1,2,3]. At the same time, AMR-associated mortality is expected to rise markedly, with estimates suggesting that annual deaths may reach several million worldwide by mid-century [4,5,6].

AMR is not limited to pathogenic bacteria. Resistance determinants are also found in commensal and environmental microorganisms, including members of the indigenous microbiota of humans, animals, and diverse environmental niches. Among these, lactobacilli are of particular interest because they are abundant in food-associated ecosystems and may contribute to the persistence and dissemination of resistance genes under both selective and non-selective pressures [7,8]. Although the taxonomy of this group has been substantially revised in recent years, lactobacilli remain ecologically versatile and widespread in plant-associated environments, animal-associated habitats, and a broad range of fermented foods [9,10,11,12].

AMR in lactobacilli has been investigated in isolates from fermented dairy products as well as from human, animal, and environmental microbiomes. However, important gaps remain. Much of the available evidence comes either from industrially produced fermented foods, where controlled starter cultures and standardized hygienic conditions may limit environmental influence, or from artisanal products examined only at the final edible stage. As a result, earlier fermentation stages, which may better reflect dynamic environmental inputs during processing and handling, have been insufficiently studied. Consequently, the extent to which fermentation stage and open-environment exposure shape the AMR phenotype of lactobacilli in artisanal dairy systems remains unclear.

Within the One Health framework, AMR emergence and dissemination are driven by interconnected reservoirs and transmission pathways linking humans, animals, food, and the environment [13,14,15]. Artisanal fermented dairy products provide a particularly relevant system for examining these interactions, because production often takes place in rural household settings with close environmental connectivity. Milk processing may occur in cellars, storerooms, or nearby livestock-associated spaces, where contact with household members, farm workers, animals, and airborne dust may facilitate microbial exchange. In such settings, stage-based sampling may help reveal whether resistance profiles shift during fermentation and handling, consistent with the acquisition, enrichment, or selection of resistance traits.

In the present study, we use a two-stage artisanal fermentation system, “Tyrovolia” (an intermediate fermented dairy matrix) transitioning to “Kopanisti” (the ripened product), as a model to test the hypothesis that artisanal fermentation stages can function as sensitive “sensors” of environmental AMR pressure. Specifically, we investigate (i) how lactobacilli populations are modified across the intermediate-to-ripened transition and (ii) whether their antimicrobial susceptibility profiles shift across stages when tested against a broad panel of antibiotics. By integrating process-stage sampling with a One Health perspective, this work aims to improve understanding of how artisanal dairy environments may influence AMR dynamics in food-associated lactobacilli and, by extension, contribute to AMR circulation across connected human–animal–environment interfaces.

## 2. Materials and Methods

In order to assess environmentally induced antimicrobial resistance (AMR), a total of 120 samples of the traditional artisanal cheese “Tyrovolia” (5–7-day curd) and 120 samples of the traditional artisanal Greek cheese “Kopanisti” (≥30-day curd) were collected from local producers directly at their facilities on the island of Mykonos, Greece. Although these cheeses are considered culinarily distinct, they originate from the same curd sampled at different time points during fermentation. “Tyrovolia” is a fresh product represented by a 5–7-day curd, whereas “Kopanisti” is produced from the remaining portion of the same curd, which is further fermented for at least 30 days with the addition of salt. In both products, according to artisanal practice, milk is not heated to pasteurization temperatures but rather to substantially lower temperatures. The two sampling points were selected to represent two distinct stages of the same artisanal fermentation process. “Tyrovolia” (5–7-day curd) corresponds to an early fermentation stage, during which the microbiota is expected to more closely reflect the original milk microbiota and animal-associated microbial inputs. In contrast, “Kopanisti” (≥30-day curd) represents a later stage, after prolonged fermentation, salt addition, and greater exposure to artisanal environmental conditions. Comparison of these two stages allowed an initial evaluation of whether antimicrobial resistance profiles change over the course of fermentation. The selected time points were defined empirically, as similar stage-based studies on these traditional products are not currently available. Under normal sampling conditions, samples were immediately placed in sterile polyethylene bags, sealed, and transported to the laboratory in ice-packed cooler boxes. Upon arrival, samples were stored at 2–4 °C and maintained under refrigeration until analysis. *Note*: Samples corresponding to the “Kopanisti” stage have been partially described in a previous publication [16]. In the present study, they are re-examined as part of an expanded dataset designed to allow comparative evaluation across production stages within a unified analytical framework. The current work therefore does not merely reproduce earlier findings but broadens the study by including additional samples and newly integrated analyses conducted under the same overall experimental design.

### 2.1. Phenotypic Identification Presumptive Lactobacilli Isolates

To isolate lactic acid bacteria (LAB), the rind of each curd was aseptically removed. From the remaining portion, 10 g was weighed and homogenized for 2 min in pre-warmed (40 °C) peptone water (10 g L^−1^ casein peptone, 5 g L^−1^ NaCl, 20 g L^−1^ trisodium citrate dihydrate; pH 7.0) using a stomacher blender. The homogenate was serially diluted and cultivated on (i) modified decarboxylating agar supplemented with histidine (MDAH) and (ii) MRS medium (de Man, Rogosa and Sharpe). After anaerobic incubation at 37 °C for 24 h, 20 µL (0.02 mL) of the appropriate dilutions were spread-plated on MRS agar and incubated anaerobically for an additional 48 h. Well-isolated colonies were selected and preliminarily screened by Gram staining and standard phenotypic assays (catalase, oxidase, and motility). Isolates meeting the criteria for presumptive LAB were subsequently subjected to species-level identification using matrix-assisted laser desorption/ionization time-of-flight mass spectrometry (MALDI-TOF MS).

Morphologically distinct, well-separated colonies were selected from each sample and subcultured by successive streaking on fresh MRS agar plates until purity was confirmed. Pure isolates were cryopreserved by suspending cells in MRS broth supplemented with 10% (*v*/*v*) sterile glycerol and stored at −80 °C for subsequent analyses.

### 2.2. Species Identification

Presumptive lactobacilli were re-streaked onto MRS agar and incubated for 24 h prior to identification. Isolates were identified by MALDI-TOF MS using the Bruker Biotyper Microflex platform (Bruker Daltonik, Bremen, Germany). Spectra were acquired according to the manufacturer’s recommendations, processed with MBT Compass software (v4.1), and matched against the Bruker reference library (BDAL, Rev. 11; 10,833 entries). For each isolate, the ten highest-ranking matches and their log(score) values (0.00–3.00) were recorded. Identification reliability was interpreted using Bruker criteria: log(score) < 1.70, unreliable; 1.70–1.99, genus-level; 2.00–2.29, probable species-level (highly probable genus-level); and ≥2.30, highly probable species-level identification. Results were confirmed in two independent experiments [17,18,19].

### 2.3. Phenotypic Antimicrobial Susceptibility Testing (AST)—Determination of Minimal Inhibitory Concentration

All 1085 isolates were included in the antimicrobial susceptibility/resistance testing, and no specific selection process was applied. Lactobacilli were not selected from a broader pool of isolates; instead, they constituted the target group of microorganisms in this study, and all recovered isolates were subjected to phenotypic susceptibility testing. This approach was based on the ecological ubiquity of lactobacilli (see Supplementary Table S7), which makes them highly relevant as potential indicators of environmental influence on the artisanal dairy matrix, including the possible carriage of resistance genes.

Minimum inhibitory concentrations (MICs) were determined by broth microdilution using 96-well microtiter plates [20,21,22]. All antimicrobial powders were purchased from Sigma-Aldrich (St. Louis, MO, USA). Test inocula were prepared from fresh cultures suspended in sterile 0.9% NaCl. The standardized suspension was subsequently diluted 1:500 in LSM broth (containing 90% Iso-sensitest broth and 10% MRS.

MICs were assessed for the following antimicrobial agents (final concentration ranges): ampicillin (0.03–64 μg mL^−1^), ampicillin/sulbactam (0.03–32 μg mL^−1^), teicoplanin (0.03–32 μg mL^−1^), clindamycin (0.03–8 μg mL^−1^), erythromycin (0.03–64 μg mL^−1^), gentamicin (0.03–500 μg mL^−1^), streptomycin (0.03–500 μg mL^−1^), chloramphenicol (0.03–256 μg mL^−1^), oxytetracycline (0.03–256 μg mL^−1^), fusidic acid (0.03–256 μg mL^−1^), metronidazole (0.25–500 μg mL^−1^), trimethoprim (0.03–500 μg mL^−1^), quinupristin/dalfopristin (0.03–16 μg mL^−1^), and vancomycin (0.03–256 μg mL^−1^). The concentration ranges of the antimicrobial agents were selected individually for each compound, taking into account their expected inhibitory ranges and, where applicable, generally accepted CLSI breakpoint frameworks. However, standardized CLSI/EUCAST interpretive criteria are not consistently available for environmental and non-clinical lactobacilli.

For each well, 50 μL of the diluted inoculum was combined with 50 μL of the corresponding antimicrobial dilution (prepared in LSM), yielding the final concentration ranges stated above. Plates were incubated anaerobically at 37 °C for 48 h, after which MIC endpoints were read as the lowest antimicrobial concentration preventing visible growth. *Escherichia coli ATCC 25922* and *Staphylococcus aureus ATCC 25923* were included as quality-control strains.

Interpretation of MIC data for lactobacilli remains challenging because widely used EUCAST clinical breakpoints and CLSI interpretive criteria are not consistently available and are not always fully compatible with the growth requirements and testing conditions of all *Lactobacillus* spp. Therefore, susceptibility categorization was based on a population-based interpretation of MIC distributions within each species, in order to distinguish the predominant wild-type (WT) population from isolates showing evidence of reduced susceptibility. Accordingly, isolates were classified as non-wild-type (NWT), indicative of acquired resistance and hereafter considered putatively resistant, when their MIC values formed a distinct subpopulation separated from the main WT cluster. Evidence for an NWT subpopulation was inferred from two distributional patterns: (i) discontinuous MIC distributions, in which one or more isolates grew only at higher MIC dilutions that were not contiguous with the modal WT range, suggesting a discrete less-susceptible group; and (ii) bimodal or shouldered distributions, in which growth was observed across consecutive MIC dilutions, but the number of isolates increased again at the upper end of the MIC range, suggesting the emergence of a second, less-susceptible cluster rather than simple tailing of the WT distribution. Because the strains were isolated from artisanal matrices exposed to environmental influences, this subpopulation-based approach was considered more appropriate than reliance on reference values developed mainly for clinical or industrial strains.

### 2.4. PCR Analysis—Detection of Antibiotic Resistance Genes

From the isolate collection, strains affiliated with the *Lactobacillus acidophilus* species group were prioritized for downstream screening of acquired antimicrobial-resistance (AMR) determinants. This group was selected because it was consistently recovered among the LAB isolates and represents a relevant lineage for assessing the potential carriage of transferable resistance genes within dairy-associated microbiota. Genotypic screening was performed using simplex PCR assays to detect resistance genes associated with four major antimicrobial classes: tetracyclines, β-lactams, chloramphenicol, and macrolides (erythromycin). These targets were chosen for three complementary reasons. First, the corresponding agents (or related compounds) are widely used in both human and veterinary practice, creating opportunities for the selection and maintenance of resistance traits across connected ecosystems. Second, these antimicrobial classes include drugs that are clinically important for human health, making the detection of resistance determinants relevant from a public-health perspective. Third, resistance genes linked to these classes are frequently located on mobile genetic elements and therefore have an increased likelihood of horizontal transfer between bacterial hosts. In the context of fermented dairy products, identifying such determinants is also informative for evaluating whether resistance signatures reflect environmental or production-chain inputs (e.g., farm, processing environment, or raw-material microbiota) rather than intrinsic properties of the LAB themselves.

PCR assays were performed to detect selected antimicrobial resistance genes associated with the phenotypes under investigation. For tetracycline resistance, two complementary genetic mechanisms were examined: ribosomal protection and active efflux. Ribosomal protection was initially screened using a universal primer set targeting ribosomal protection determinants and, in positive isolates, was further verified by amplification with *tet*M-specific primers in order to identify the determinant at the gene level. Active efflux was assessed by detection of the *tet*K gene, which encodes a major facilitator superfamily (MFS)-type tetracycline efflux pump. In addition, the presence of *bla*TEM, *cat*, and *ermB* was investigated as markers of resistance to β-lactams, chloramphenicol, and macrolides, respectively. More specifically, *bla*TEM encodes a β-lactamase capable of hydrolyzing β-lactam antibiotics, *cat* encodes chloramphenicol acetyltransferase responsible for enzymatic drug inactivation, and *erm*B encodes a 23S rRNA methyltransferase that mediates target-site modification and is commonly associated with the MLS_B_-type resistance phenotype. Primer sequences, expected amplicon sizes, and PCR cycling and annealing conditions, together with the corresponding methodological references [23,24,25,26,27,28,29,30], are presented in Supplementary Table S1.

Genomic DNA was prepared from freshly grown colonies to minimize DNA degradation and to ensure consistent template quality. Briefly, a single well-isolated colony from each isolate was harvested and processed using the DNeasy^®^ UltraClean^®^ Microbial Kit (QIAGEN) according to the manufacturer’s instructions. Purified DNA was eluted as provided by the kit protocol and stored at −20 °C until further analysis. A no-template control (NTC; RNase-free water in place of DNA) was included in every run to control for reagent contamination and carry-over during reaction setup. Amplification was performed on a CFX96™ thermocycler (Bio-Rad, Hercules, CA, USA). PCR products were separated on 2% (*w*/*v*) agarose gels at 100 V, stained, and visualized, and fragment sizes were estimated by comparison with an appropriate DNA molecular weight marker. The PCR cycling conditions consisted of an initial denaturation at 94 °C for 5 min, followed by 35 cycles of denaturation at 94 °C for 30 s, annealing at the primer-specific temperature (Supplementary Table S1) for 30 s, and extension at 72 °C for 45 s. A final extension step at 72 °C for 7 min completed the protocol.

### 2.5. Questionnaire Survey on Farm Antimicrobial Use

Prior to sampling, the attending veterinarian administered a brief structured questionnaire to the participating producers in order to document routine antimicrobial use in their sheep flocks. The survey recorded the most common clinical indications for treatment, the estimated frequency of antimicrobial administration, and the antibiotic agents and routes of administration most commonly used for therapeutic purposes.

### 2.6. Statistical Analysis Section

Chi square test of independence was used to evaluate changes in the number of resistant strains during fermentation. The Pearsons correlation coefficient test was used to assess various correlations (e.g., the number of AMR strains to MDR strains and others). Relative Risk of AMR was calculated for each antibiotic as well as for every species (wherever possible). Fischer’s exact test was used for the assessment of the combined analysis of phenotypic resistance and corresponding resistance genes in *L. acidophilus* isolates. Multilinear regression was used to determine predictors for AMR and MDR. In all tests significance was considered for *p* < 0.05.

## 3. Results

Table 1 summarizes the antimicrobial panel used in this study, organized by antibiotic class, primary cellular target/mode of action, and the major resistance mechanisms typically associated with each compound. This framework is used throughout the Results to support interpretation of the susceptibility profiles observed in lactobacilli isolates across the “Tyrovolia”-to-“Kopanisti” transition. In particular, grouping antibiotics by shared targets (cell-wall synthesis, protein synthesis, nucleic-acid integrity, and folate metabolism) allows the resistance phenotypes presented in the subsequent tables to be read not merely as isolate-by-isolate outcomes, but as mechanistically coherent patterns—for example, phenotypes consistent with β-lactamase activity or altered penicillin-binding proteins (β-lactams), target-site modification and efflux (macrolides/lincosamides), efflux- and ribosomal protection–associated tetracycline resistance, aminoglycoside-modifying enzymes, or glycopeptide-associated peptidoglycan precursor remodeling. Accordingly, Table 1 serves as a reference map for the stage-dependent shifts in antimicrobial susceptibility reported below and helps contextualize potential routes of resistance acquisition, enrichment, or selection within an artisanal, environmentally exposed fermentation system.

Table 2 summarizes the producers’ responses regarding routine therapeutic antibiotic use in their sheep flocks, including the most frequently reported infection categories, their estimated prevalence, and the antimicrobial agents commonly administered. Respiratory infections were reported as the most frequent condition (4–5%), typically treated with tetracycline via subcutaneous (SC) or intramuscular (IM) administration. Udder, post-partum, and traumatic infections were reported at lower frequencies (1–3%) and were consistently managed with a penicillin/dihydrostreptomycin combination administered intramuscularly. Overall, the interview data indicate that the on-farm therapeutic antimicrobial exposure relevant to this study was dominated by tetracyclines and β-lactam/aminoglycoside combination therapy, providing contextual background for the susceptibility profiles of lactobacilli isolates presented in the subsequent sections.

Table 3 provides an overview of the lactobacilli isolated from the two fermentation stages examined in this study and summarizes, by species, the frequency of resistant phenotypes and multidrug resistance. In total, 518 isolates were recovered from the 5-day curd (“Tyrovolia”) and 567 isolates from the 30-day curd (“Kopanisti”), comprising 16 species. Resistance phenotypes were detected across the antimicrobial panel; however, resistance was not uniformly distributed among species. Notably, all *L. johnsonii* and *L. casei* subsp. *pseudoplantarum* isolates from the 5-day curd were susceptible to all tested antibiotics, and the same complete susceptibility was observed for *L. casei* subsp. *pseudoplantarum* isolates recovered from the 30-day curd. Overall, the proportion of resistant isolates was significantly higher in the 30-day curd (438/567; 77.25%) than in the 5-day curd (362/518; 69.88%) (χ^2^ = 7.58, *p* = 0.0059). In addition, multidrug-resistant (MDR) isolates (resistance to >2 antibiotic classes) were detected at a significantly higher frequency in the 30-day curd (266/438; 60.73% of resistant isolates) compared with the 5-day curd (138/362; 37.57%) (χ^2^ = 40.53, *p* < 0.001). Together, these findings indicate a stage-associated increase in both overall resistance and MDR phenotypes during the transition from the intermediate curd to the ripened product.

Table 4 breaks down multidrug resistance (MDR) at the species level and shows which exact antibiotics each MDR isolate resisted. In contrast to Table 3, which summarizes MDR counts, Table 4 lists the specific resistance combinations observed within each *Lactobacillus* species and indicates how many distinct MDR phenotypes occurred per species. This format allows a direct comparison between the 5-day and 30-day curds to determine whether the higher MDR frequency at day 30 is driven by (i) more MDR isolates within the same species, (ii) a broader range of MDR profiles within species, or (iii) different species contributing disproportionately to MDR.

Table 5 summarizes, for each of the 14 antibiotics tested, the number (and percentage) of resistant lactobacilli isolates recovered from the 5-day and 30-day curds and quantifies stage-associated change using relative risk (RR) with 95% confidence intervals (CI). No significant stage effect was observed for clindamycin, gentamicin, or fusidic acid, as the RR values were close to unity and the corresponding CIs included 1. In contrast, resistance to erythromycin, and oxytetracycline decreased significantly in the 30-day curd (RR < 1), indicating a lower probability of detecting resistant isolates at the ripened stage. For the remaining nine antibiotics, resistance increased significantly during the transition from the 5-day to the 30-day curd (RR > 1), consistent with stage-associated enrichment and/or acquisition of resistance phenotypes under artisanal, environmentally exposed conditions. The strongest increases were observed for metronidazole and chloramphenicol, followed by quinupristin/dalfopristin, which exhibited the highest RR values and narrow CIs that did not overlap 1.

Table 6 presents the range of MIC values, the MIC50 and MIC 90 values as well as the experimental cut-off values of *L. acidophilus* isolates from the 5th and the 30th day matrix. In general, most values of MIC 50 and MIC90 increased under the environmental impact.

In Table 7 the detected genes are depicted with the relevant phenotypes. There is a distinct difference between the expression of these genes between the 5th and the 30th day matrixes.

The combined analysis of antimicrobial susceptibility and PCR-based gene detection in *L. acidophilus* revealed only partial agreement between phenotypic resistance and the investigated resistance determinants (Table 8). While phenotypic resistance was observed for multiple antibiotics, the corresponding genes were not uniformly detected among resistant isolates. More specifically, the genes *bla*TEM, *erm*B, *tet*M, *tet*K, and *cat* were present in only a proportion of isolates exhibiting resistance to the related antibiotic classes, indicating incomplete genotype–phenotype correspondence. This pattern may reflect the involvement of additional, non-investigated resistance genes, mutations affecting target sites, regulatory effects on gene expression, or other intrinsic and acquired resistance mechanisms. Therefore, the molecular screening performed herein supports the phenotypic findings only in part and highlights the complexity of antimicrobial resistance in *L. acidophilus* isolates.

To further substantiate the findings of the present study, the detailed antimicrobial susceptibility data are provided in the Supplementary Materials, where they provide a more granular view of the resistance patterns observed among the isolated *Lactobacillus* strains. Specifically, Tables S2 and S3 report the MIC ranges and the corresponding experimental cut-off values for isolates recovered on the 5th and 30th day, respectively, thus allowing direct evaluation of how susceptibility profiles shifted across the two fermentation stages. Building on this comparison, Table S4 summarizes changes in the upper limits of the MIC ranges between day 5 and day 30, thereby highlighting the direction and extent of these shifts and facilitating the identification of antibiotics for which resistance trends became more pronounced during ripening. In parallel, Table S5 presents the EFSA (2018) cut-off values for the relevant *Lactobacillus* groups and species, providing the regulatory benchmark against which the present data can be interpreted [31]. Finally, Table S6 compares the number of resistant strains classified according to EFSA breakpoints with those classified using the experimental cut-off values established in this study, thereby illustrating the degree to which resistance estimates depend on the interpretive framework applied. Taken together, these supplementary tables do not merely provide additional raw data but substantively reinforce the main results by documenting the stage-dependent dynamics of antimicrobial susceptibility and by showing that the apparent prevalence of resistance may vary according to the criteria used for classification.

## 4. Discussion

A central premise of the present work is that artisanal, open-environment dairy fermentations operate at a One Health interface, where microorganisms can be exchanged among livestock, humans, companion animals, and surrounding environmental niches during routine handling and ripening. Accordingly, interpretation of stage-dependent shifts in the lactobacilli community—and in associated antimicrobial resistance phenotypes—should consider not only “starter-like” dairy-adapted taxa, but also taxa plausibly introduced from, or selectively enriched by, the broader production environment. To support this ecological interpretation, Table 6 compiles the lactobacilli taxa isolated in the present study and summarizes their reported occurrence across potential reservoirs described in the literature, including livestock-associated sites (gastrointestinal tract, oral cavity, udder, skin, vagina, nasal cavity), human-associated niches (skin, oral cavity, gastrointestinal tract, nasal cavity), companion animals, and abiotic matrices such as vegetation/rhizosphere and soil. This synthesis is not intended to attribute a definitive origin to any isolate, because species-level occurrence across multiple niches limits source inference based on taxonomy alone, but rather to provide a biologically plausible framework for understanding how environmental exposure during the “Tyrovolia”-to-“Kopanisti” transition could reshape microbial load and community composition, and thereby influence the resistance landscape through introduction, persistence, and/or selective enrichment of specific taxa. In this context, Supplementary Table S7 serves as an interpretive “road map”, helping to contextualize the observed stage-dependent patterns in microbial composition and antimicrobial resistance within a realistic One Health framework.

Resistance of lactobacilli to antibiotics has been widely investigated owing to the genus’s ubiquitous presence in nature and, above all, its extensive application as a probiotic in animal and human nutrition [32,33,34,35]. Intrinsic, or innate, resistance is generally attributed to chromosomal determinants, as demonstrated in glycopeptide resistance, including vancomycin resistance, in *L. sakei*, *L. plantarum*, and *L. rhamnosus* [36,37]. By contrast, acquired resistance is typically linked to mobile genetic elements, such as plasmids and transposons, whose horizontal transfer can give rise to resistant strains and promote the further spread of resistance [38]. Intrinsic resistance in lactobacilli has also been documented for trimethoprim [8], aminoglycosides (gentamicin and streptomycin), metronidazole, and fluoroquinolones [21,39,40,41].

However, a notable gap in the scientific literature is the investigation of environmentally exposed lactobacilli and their capacity to adapt to environmental conditions that may favor increased antimicrobial resistance. Intensive farming represents a typical environment of this kind because of its extensive use of antibiotics, although it is by no means the only one. Artisan production may also provide prolonged environmental exposure, as these products interact more directly with their surroundings, unlike industrial production, where fermentation processes are generally carried out under controlled conditions. In artisanal settings, products may be exposed to humans (visitors or residents), roaming pets or farm poultry, and fluctuations in weather conditions. Temporary overcrowding within a limited area may likewise contribute to the dissemination of resistance, particularly when combined with heavy foot traffic in animal-rearing or artisanal production premises. Collectively, these factors may increase the likelihood that lactobacilli acquire resistance determinants or adapt through elevated minimum inhibitory concentrations (MICs).

Our findings suggest that environmental exposure significantly affects the resistance profiles of lactobacilli. As shown in Tables S1 and S2, the upper limits of the MIC ranges increased significantly from day 5 to day 30, regardless of whether discrete subpopulations were present or emerged within a species. Of the 224 possible combinations of *Lactobacillus* species and antibiotics (16 species × 14 antibiotics), 129 (57.58%) showed an increase in the upper limit of the MIC range at day 30, 33 (14.73%) showed a decrease, and 58 (25.89%) remained unchanged. This overall trend is clearly presented in Table S3. The effect was not uniform across species (*p* < 0.001). For example, *L. helveticus* and *L. curvatus* showed increases in the upper limit of the day-30 MIC range for 12 of the 14 antibiotics tested, whereas *L. pentosus* showed decreases for 6 antibiotics, no change for 2, and increases for the remaining 6. Likewise, the effect varied significantly among antibiotics (*p* < 0.001). Vancomycin, for instance, showed an increase in the upper limit of the MIC range in 15 of the 16 *Lactobacillus* species, whereas penicillin G showed a decrease in 7 species and an increase in 8 species. These observations underline the existence of a species–antibiotic-specific relationship. To some extent, this relationship appeared to extend beyond the phenomenon of innate resistance. Even in the case of vancomycin, which is classically associated with intrinsic resistance in certain *Lactobacillus* species, the upper limit of the MIC range still increased in the day-30 matrix for those species. In Table 3 and Table 4 the relation between the resistant strains, the multiresistant ones, the phenotypes and the classes of antibiotics against which resistance was recorded, can be seen. This relationship proved to be strongly positive (R ≥ 0.774, *p* < 0.001) for the various combinations of these variables. Multilinear analysis however showed that the only significant predictor for MDR strains in the 5th day matrix was the number of phenotypes (R = 0.857, *p* < 0.001) while for MDR in the 30th day matrix was the number of resistant strains (R = 0.954, *p* < 0.001). As a matter of fact, 38.12% of the resistant strains of the 5th day matrix were found MDR, while 60.73% of the 30th day matrix were found MDR (OR = 2.51, CI 95% 1.886–3.341, *p* < 0.001) which practically means that every resistant strain isolated from the 30th day matrix has 2.51 more chances to be MDR than the ones of the 5th day matrix and this finding implies strong impact of the environmental exposure.

Interestingly, resistance was not universal among the lactobacilli examined. A substantial proportion of isolates remained susceptible to all antibiotics tested, accounting for 30.12% of isolates recovered from the day-5 matrix and 22.75% of those recovered from the day-30 matrix. As this difference was not statistically significant, these findings may indicate the persistence of a baseline susceptible subpopulation throughout the experimental period. One possible explanation is the existence of competitive interactions among resistant phenotypes, whereby strains resistant to one antimicrobial may be outcompeted by strains carrying resistance to another. This interpretation may also help explain why five antibiotics yielded a relative risk (RR) below 1. For example, lactobacilli resistant to ampicillin may have been outcompeted by strains resistant to the sulbactam/ampicillin combination, while gentamicin-resistant isolates may have been displaced by streptomycin-resistant counterparts. Although this hypothesis requires further investigation, it suggests that shifts in resistance prevalence may not depend solely on direct selection pressure, but also on ecological interactions and fitness trade-offs within the lactobacillus community.

In the present study, strains were classified as resistant when they formed a clearly distinct subpopulation within isolates of the same species, whereas the highest MIC value observed for the susceptible subpopulation was taken as the experimental cutoff. The results of this classification approach are presented in Table 3 and Tables S2 and S3. In 2018, EFSA established breakpoint values for *Lactobacillus* species for nine antibiotics, eight of which were included in the present study (Table S5) [31]. Because the EFSA breakpoints are higher than the experimental cutoffs derived here, applying the EFSA criteria to our dataset results in substantially fewer strains being categorized as resistant (Table S6).

Nevertheless, even under the EFSA breakpoint framework, the number of resistant strains recovered from the day-30 matrix remained higher than that isolated from the day-5 matrix, further supporting the conclusion that environmental exposure promotes the emergence or enrichment of resistant phenotypes. This observation is particularly important, as it indicates that the environmental effect identified in the present study is robust and does not depend solely on the stricter classification criteria adopted here. In other words, although our experimental cutoffs were more conservative than the currently accepted EFSA thresholds, the same overall pattern remained evident when the official breakpoints were applied. EFSA breakpoints were developed primarily for practical and regulatory purposes, including the safety assessment of probiotic strains intended for animal feed and human consumption. Although some authors have suggested that current EFSA microbiological cutoffs should be re-examined upward for certain *Lactobacillus* species–antibiotic combinations, our findings point in the opposite direction. Specifically, the present data suggests that higher breakpoint values may underestimate the proportion of strains with reduced susceptibility and may therefore obscure early shifts in MIC distributions associated with environmental exposure. From this perspective, lower or more sensitive microbiological cutoffs may better capture the adaptive dynamics of lactobacilli under environmentally relevant conditions [8,31,42,43].

*L. acidophilus* was selected for molecular analysis of resistance determinants because it was the most prevalent species among the isolates, representing 70 isolates (13.51%) in the day-5 matrix and 71 isolates (12.52%) in the day-30 matrix. In addition, the number of resistant strains was strongly correlated with the total number of isolates (R = 0.95, *p* < 0.0001 for the day-5 matrix; R = 0.949, *p* < 0.001 for the day-30 matrix), indicating that this species provided the greatest likelihood of detecting resistance genes. The frequencies of the detected genes are presented in Table 7 and Table 8. A total of 21 genes were detected in 14 strains from the day-5 matrix, whereas 19 genes were detected in 13 strains from the day-30 matrix. More importantly, clear genotype–phenotype discordance was observed between the two matrices. The *tet*M gene was associated with oxytetracycline resistance in strains from the day-5 matrix, but with phenotypic susceptibility in strains from the day-30 matrix. A similar pattern was found for *erm*B, which corresponded to erythromycin resistance in the day-5 matrix but to susceptibility in the day-30 matrix. Likewise, *bla*TEM was associated in the day-5 matrix with resistance only to ampicillin, whereas in the day-30 matrix it was associated with resistance to all three β-lactam antibiotics tested. These findings indicate that environmental exposure may affect not only the occurrence of resistance determinants but also their expression. The presence of a resistance gene did not consistently translate into phenotypic resistance, and this relationship appeared to depend on the environmental matrix. Similar genotype–phenotype discordance has been reported previously. Hütt et al. (2018) identified *tet*M and *tet*K in lactobacilli that remained susceptible to oxytetracycline [44], while Anisimova et al. (2019) described strains carrying *tet*K and *erm*B despite showing susceptible phenotypes [37]. Such evidence suggests that molecular detection alone is insufficient for reliable prediction of resistance expression. Several mechanisms may explain this discrepancy. Resistance genes may be transcriptionally silent, altered by mutations in regulatory regions, or inactivated by premature stop codons, insertions, or deletions [45,46]. Even so, non-expressed genes may still remain epidemiologically relevant because they can retain the potential for horizontal transfer [47]. This is particularly important in environmentally exposed systems, where opportunities for gene exchange may be enhanced even in the absence of immediate phenotypic expression.

The detected genes also differed in function and mobility. *tet*M encodes a ribosomal protection protein and may be located on either chromosomes or plasmids, whereas *tet*K encodes an efflux pump and is most often plasmid-borne, increasing its transfer potential [8,38]. *erm*B encodes an rRNA methylase targeting the 23S ribosomal subunit and is generally chromosomal, although it remains a key determinant of macrolide resistance [29,48]. The cat genes encode chloramphenicol acetyltransferases and are frequently associated with mobile genetic elements such as plasmids, supporting their transferability [49]. Finally, *bla*TEM genes are well known for their high horizontal mobility within bacterial communities [50]. Taken together, these results suggest that environmental exposure may shape antimicrobial resistance in lactobacilli through mechanisms extending beyond simple gene acquisition. In addition to selecting resistance determinants, environmental conditions may influence whether such genes are phenotypically expressed. This highlights the complexity of resistance ecology in lactobacilli and underscores the need to interpret molecular findings alongside phenotypic susceptibility data. The observed changes in resistance patterns do not, by themselves, identify the exact mechanism responsible for these shifts. Although environmental exposure is considered an important factor, especially under artisanal production conditions with limited protection from external microbial inputs, other explanations should also be taken into account. These include microbial competition, ecological selection during fermentation and ripening, and possible horizontal transfer of resistance determinants within bacterial communities. Therefore, the term environmental pressure should be interpreted broadly, encompassing not only direct exposure to environmental microbiota, but also the selective and interactive processes operating during fermentation. Given the open and dynamic nature of artisanal production environments, the observed AMR shifts are likely to reflect the combined effects of environmental exposure, microbial interactions, and fermentation-associated selection rather than a single causal mechanism [51,52].

## 5. Limitations of This Study

A limitation of the present study lies in the selection of only two fermentation stages to assess antimicrobial resistance dynamics during artisanal cheese production. These time points were chosen to represent two distinct phases of the process and to allow an initial evaluation of resistance patterns over time in relation to environmental exposure. The selection was empirical, as closely comparable stage-based studies were not available. In this context, the 5–7-day curd may still reflect, to a considerable extent, the original milk microbiota and animal-associated inputs, whereas the ≥30-day curd is more likely to reflect the cumulative effects of fermentation, handling, and environmental exposure. Nevertheless, inclusion of additional intermediate sampling points would provide a more refined view of the temporal evolution of AMR during fermentation and should be considered in future investigations.

This consideration also relates to a broader interpretive constraint of the study. Comparisons with previous reports should be made with caution, since differences in artisanal production practices, fermentation stage, microbial ecology, and analytical methodology may substantially influence the AMR patterns observed in lactobacilli. Accordingly, although the present findings offer useful insight into the possible dynamics of resistance in artisanal dairy systems, they should be interpreted within the specific ecological and technological context of the production system examined here.

## 6. Conclusions

The present study demonstrates that artisanal dairy matrices sampled at different fermentation stages provide a useful model for investigating antimicrobial resistance (AMR) in environmentally exposed lactobacilli. The novelty of the present research does not rely upon the isolation of resistant bacteria from artisan products but on the recording of this resistance in two different time frames and thus proving that resistance is a dynamic not static effect and -particularly for artisan products- reflects the environmental impact. In that sense artisan products can be used as a sentinel system for assessing the environmental burden of AMR. In industrial scale fermentations, the environment in which these fermentations take place is highly monitored by systems checking the air, the circulation of personnel, etc., and thus its impact is minimal as it should be otherwise the fermented products could end with different properties in every lot, depending on the circumstances. Resistance increased overall from day 5 to day 30 across most *Lactobacillus* species and for most antibiotics tested, while molecular analysis revealed the presence of resistance determinants, including genes with transfer potential, as well as genotype–phenotype discordance. These findings suggest that prolonged environmental exposure during artisanal fermentation may contribute not only to the selection or enrichment of resistant phenotypes, but also to variation in the expression of resistance genes. From a One Health perspective, artisanal dairy fermentations may therefore serve as practical indicators of local AMR pressure at the interface of food, animals, humans, and the environment. Further studies integrating phenotypic, genomic, and source-tracking approaches are needed to clarify the mechanisms underlying these shifts and their epidemiological significance.

In this study we propose a paradigm: monitoring the artisan matrix for resistant lactobacilli in two different time frames and assessing the observed difference, to conclude on environmental burden of resistance genes. This paradigm can work in any artisan environment, since in artisan production the controls of industrial production are not applicable.

## Figures and Tables

**Table 1:** Antimicrobial agents included in the susceptibility testing panel of the present study, grouped according to antimicrobial class, principal mode of action, and major reported resistance mechanisms.

Substance	Class	Mode of Action	Mechanisms of Resistance
Ampicillin	β-lactams	Binds to enzymes involved in peptidoglycan synthesis, inhibiting cell wall formation	-β-lactamases inactivate the antibiotic -changes in cell wall protein enzymes
Sulbactam	β-lactamase inhibitor	Inhibits the β-lactamase enzymes	Extended spectrum β-lactamases (classes A–D)
Erythromycin	Macrolides	Binds to the 23S rRNA molecule of the 50S ribosomal subunit and blocks protein synthesis by inhibiting the transpeptidation/translocation step and also by inhibiting the assembly of the 50S subunit	-target site modification by methylation of a specific nucleotide of the 23S rRNA (*erm* genes) -active drug efflux (*mef*A, *mef*E genes) -cross resistance via the MSLB phenotype
Clindamycin	Lincosamides	-prevents peptide bond formation by binding to the 23S rRNA molecule of the 50S ribosomal subunit. It impedes the assembly of the subunit as well as the translation process.	-target modification: (i) mutation in the 23S rRNA (ii) ribosomal methylations (*erm* genes) -active efflux -target protection by specific proteins -antibiotic inactivation by specific enzymes
Oxytetracycline	Tetracyclines	Binds to the 30S ribosomal subunit and interferes with amino acid transfer	-Inducible efflux -binding site change
Chloramphenicol	Phenicols	Binds to the L16 protein of the 50S ribosomal subunit and inhibits the elongation of peptide chains by suppressing the peptidyl transferase.	-Enzymatic inactivation: (i) CATs: chloramphenicol acetyltransferases (ii) CPTs: chloramphenicol phosphotransferases -efflux pumps -permeability barriers by alterations in outer membrane proteins -target site mutations
Gentamycin	Aminoglycosides	Binds to the 30S ribosomal subunit causing misread of code and thus disrupting membrane permeability and the production of mistranslation of proteins	-Phosphorylation -Adenylation -Acetylation
Streptomycin		Inhibition of protein synthesis at the aminoacyl transfer site of the 16S part of the 30S ribosomal subunit. It binds the formyl -methionyl-tRNA to the 30S subunit.	-Enzymatic inactivation (genes encoding such enzymes are *str*A and *str*B) -mutations in genes encoding ribosomal proteins lead to alterations in the ribosomal subunit (*rps*L and *rrs* genes) -changes in membrane permeability -efflux pumps
Vancomycin	Glycopeptides	Bind to the D-Ala-D-Ala terminus of peptidoglycan precursors preventing cell wall synthesis.	Modification of the bacterial cell wall’s peptidoglycan structure: the terminal Alanine is replaced by other dipeptides Serine or Lactate: D-Ala-D-Ser or D-Ala-D-Lac (*van*A and *van*B gene clusters and VanC protein)
Teicoplanin			-As vancomycin above -mutations in the genes encoding D-Ala-D-Ala ligase -VanJ novel protein of the bacterial cell wall reduces the affinity of the drug to its target
Fusidic acid	Tetracyclic steroid (fusidanes)	Binds to the elongation factor G (EF-G) on the ribosome preventing the translocation of the peptide chain inhibiting thus the protein synthesis.	-mutation in the *fus*A gene which encodes EF-G and/or mutation in the *fus*E gene which encodes the ribosomal protein L6 -efflux pumps -enzymatic inactivation -altered drug permeability
Metronidazole	Nitroimidazoles	After intracellular reduction of its molecule, metronidazole interacts with DNA causing strand rupture, helix destabilization and cell death	-reduced uptake or increased efflux -increased DNA repair -altered pyruvate metabolism (necessary to the anaerobic reduction of the drug) -mutation in genes like *rdx*A encoding proteins involved in the activation pathways of metronidazole
Quinupristin/dalfopristin	Streptogramins	-bind on different sites of the 50S ribosomal subunit: Dalfopristin inhibits the early phase of protein synthesis while Quinupristin inhibits the late phase.	-enzymatic inactivation, e.g., acetyltransferases can inactivate Dalfopristin (genes *vat*A, *va*tB and *vat*C). -active efflux (genes *vga*A and *vga*B) -target modification
Trimethoprim	Diaminopyridine	Inhibits dihydrofolate reductase (DHFR), an enzyme necessary for folic acid synthesis in the bacterial cell. Folic acid is crucial to bacterial DNA and protein synthesis.	-mutations in DHFR genes (*dfr*)leads to synthesis of altered DHFR molecule resistant to trimethoprim.

**Table 2:** Results of interviews with farm owners regarding the use of antibiotics for common infections in livestock.

Most Common Infections	Prevalence Rate (%)	Antibiotics Used Systematically for Therapeutic Purposes
Respiratory	4–5	Tetracycline SC or IM
Udder	1–2	Penicillin/Dihydrostreptomycin IM
Post partum	2–3	Penicillin/Dihydrostreptomycin IM
Traumatic	1–2	Penicillin/Dihydrostreptomycin IM

Abbreviations: SC, subcutaneous; IM, intramuscular.

**Table 3:** Species-level distribution of lactobacilli isolates and antimicrobial resistance outcomes across fermentation stages (5–7-day “Tyrovolia” curd vs. ≥30-day “Kopanisti” curd).

Species	5th Day Curd					30th Day Curd				
	n ^a^	R ^b^	A/B ^c^	Ph/T ^d^	MDR ^e^	(n)	R	A/B	Ph/T	MDR
*L. helveticus*	19	13	0	0	0	31	27	4	3	7
*L. acidophilus*	70	48	6	6	36	71	66	6	7	43
*L. sakei*	43	33	4	3	15	24	19	5	4	7
*L. paraplantarum*	48	48	8	10	35	48	43	8	8	37
*L. brevis*	12	7	3	2	5	30	11	2	2	0
*L. delbrueckii subsp bulgaricus*	58	47	7	8	17	84	80	7	11	54
*L. johnsonii*	19	0	0	0	0	49	26	4	3	19
*L. curvatus*	36	30	3	3	0	42	33	7	6	30
*L. salivarius*	19	11	1	1	0	12	12	5	3	0
*L. plantarum*	36	18	2	1	0	54	47	6	6	36
*L. rhamnosus*	38	19	1	1	0	30	24	6	4	13
*L. delbrueckii subsp lactis*	48	39	5	6	12	36	18	4	3	6
*L. fermentum*	24	17	6	3	6	36	19	3	3	7
*L. pentosus*	29	24	8	4	12	25	7	3	1	7
*L. casei subsp casei*	13	8	2	2	0	12	6	1	1	0
*L. casei subsp pseudoplantarum*	6	0	0	0	0	6	0	0	0	0
Total	518	362	-	-	138	567	438	-	-	266

^a^: n, number of isolates; ^b^: R, number of resistant isolates; ^c^: A/B, number of antibiotic classes in which resistance was recorded for the isolates of each species, indicating the range of antimicrobial categories involved; ^d^: Ph/T, number of distinct resistance phenotypes observed within each species, defined as different combinations of susceptibility/resistance profiles among the isolates; ^e^: MDR, multidrug-resistant isolates (resistance to >2 antibiotic classes). Totals refer to all isolates recovered from the 5-day (“Tyrovolia”) and 30-day (“Kopanisti”) curds.

**Table 4:** Species-specific multidrug-resistant (MDR) phenotypes of lactobacilli recovered from the 5-day “Tyrovolia” curd and the ≥30-day “Kopanisti” curd.

Species	5-Day Curd	30-Day Curd
*L. helveticus*	**0 strains**	6 × Chlor-Met-Q/D-Oxy (4) ^a^ 1 × Chlor-Met-Q/D (3) **7 strains(22.58%)/2 phenotypes/4 antibiotics**
*L. acidophilus*	12 × Amp-Ery-Oxy-Gen-Met (5) 17 × Amp-Ery-Oxy (3) 6 × Amp-Ery-Oxy-Gen-Met (5) 1 × Amp-Ery-Oxy-Gen (4) **36 strains (51.43%) ^b^/4 phenotypes/6 antibiotics**	12 × Amp-Sulb/Amp-Chlor-Van-Met-Tri (5) 24 × Amp-Sulb/Amp-Van-Met-Tri (4) 7 × Amp-Sulb/Amp-Met-Tri (3) **43 strains(60.56%)/3 phenotypes/6 antibiotics**
*L. sakei*	15 × Amp-Clin-Chlor (3) **15 strains (34.88%)/1 phenotype/3 antibiotics**	5 × Chlor-Tri-Pen-Fus-Amp (5) 2 × Chlor-Tri-Amp (3) **7 strains(29.17%)/2 phenotypes/5 antibiotics**
*l. paraplantarum*	6 × Amp-Ery-Tri-Fus (4) 6 × Amp-Sulb/Amp-Ery-Tri-Fus (4) 1 × Clin-Ery-Tri (3) 3 × Clin-Ery-Amp (3) 2 × Clin-Ery-Amp-Sulb/Amp (3) 17 × Clin-Ery-Chlor (3) **35 strains (72.91%)/6 phenotypes/8 antibiotics**	17 × Ery-Clin-Chlor-Str-Van-Tei-Met-Fus (7) 3 × Ery-Clin-Chlor-Str-Van-Tei-Met (6) 9 × Ery-Clin-Chlor-Str-Van-Tei (5) 6 × Ery-Clin-Van-Tei (4) 1 × Ery-Fus-Van-Chlor (4) 1 × Clin-Str-Tei-Met (4) **37 strains (77.08%)/6 phenotypes/7 antibiotics**
*L. brevis*	5 × Str-Fus-Tri (3) **5 strains (41.67%)/1 phenotype/3 antibiotics**	**0 strains**
*L. delbrueckii* subsp. *bulgaricus*	10 × Amp-Ery-Oxy-Q/D (4) 6 × Amp-Ery-Oxy-Tri-Q/D (5) 1 × Amp-Ery-Oxy-Q/D (4) **17 strains (29.31%)/3 phenotypes/5 antibiotics**	23 × Amp-Chlor-Gen-Van-Met-Q/D (6) 1 × Amp-Chlor-Van-Q/D (4) 1 × Amp-Gen-Met (3) 11 × Chlor-Van-Q/D (3) 7 × Amp-Chlor-Gen-Van-Q/D (5) 5 × Amp-Chlor-Gen-Van-Q/D (5) 6 × Chlor-Van-Gen-Q/D (4) **54 strains (64.29%)/7 phenotypes/7 antibiotics**
*L. johnsonii*	**0 strains**	19 × Amp-Chlor-Str-Met (4) **19 strains (38.78%)/ 1 phenotype/4 antibiotics**
*L. curvatus*	**0 strains**	10 × Oxy-Met-Amp-Van (4) 2 × Oxy-Met-Amp-Q/D (4) 7 × Oxy-Met-Amp (3) 11 × Amp-Oxy-Met (3) **30 strains (71.43%)/4 phenotypes/5 antibiotics**
*L. salivarius*	**0 strains**	**0 strains**
*L. plantarum*	**0 strains**	30 × Amp-Clin-Tei-Met-Fus-Q/D (6) 3 × Amp-Tei-Met-Fus-Q/D (5) 1 × Tei-Met-Fus-Q/D (4) 2 × Tei-Met-Fus (30 **36 strains (66.67%)/4 phenotypes/6 antibiotics**
*L. rhamnosus*	**0 strains**	6 × Amp-Sulb/Amp-Clin-Chlor-Gen-Van-Met (6) 6 × Amp-Sulb/Amp-Clin-Chlor-Gen (4) 1 × Amp-Sulb/Amp-Chlor-Gen **13 strains(43.33%)/3 phenotypes/6 antibiotics**
*L. delbrueckii* subsp. *lactis*	6 × Amp-Sulb/Amp-Oxy-Gen-Fus (4) 6 × Amp-Sulb/Amp-Gen-Fus (3) **12 strains (25.00%)/2 phenotypes/5 antibiotics**	6 × Amp-Oxy-Gen-Met (4) **6 strains (16.67%)/ 1 phenotype/4 antibiotics**
*L. fermentum*	6 × Amp-Sulb/Amp-Str-Van-Fus (4) **6 strains (25.00%)/1 phenotype/6 antibiotics**	7 × Oxy-Str-Q/D (3) **7 strains (28.00%)/1 phenotype/3 antibiotics**
*L. pentosus*	9 × Amp-Sulb/Amp-Ery-Clin-Oxy-Str-Van-Tri (7) 1 × Amp-Sulb/Amp-Ery-Clin-Oxy-Van-Tri (6) 2 × Amp-Sulb/Amp-Ery-Oxy-Van-Tri (5) **12 strains (33.33%)/3 phenotypes/8 antibiotics**	7 × Chlor-Tri-Met (3) **7 strains (28.00%)/1 phenotype/3 antibiotics**
*L. casei* subsp. *casei*	**0 strains**	**0 strains**
*L. casei* subsp. *pseudoplantarum*	**0 strains**	**0 strains**

Notes: ^a^: indicates the number of isolates expressing each MDR phenotype × the corresponding phenotype, with the number in parentheses indicating the number of antibiotic classes involved; ^b^: indicates the percentage of MDR isolates relative to the total number of isolates of the corresponding species at that fermentation stage; Abbreviations (antibiotics): Amp, ampicillin; Sulb/Amp, sulbactam/ampicillin; Ery, erythromycin; Clin, clindamycin; Oxy, oxytet-racycline; Chlor, chloramphenicol; Gen, gentamicin; Str, streptomycin; Van, vancomycin; Tei, teicoplanin; Fus, fusidic acid; Met, metronidazole; Q/D, quinupristin/dalfopristin; Tri, trimethoprim; MDR, multidrug-resistant.

**Table 5:** Antibiotic-specific resistance frequencies in lactobacilli at the early and ripened fermentation stages (5–7-day “Tyrovolia” curd vs. ≥30-day “Kopanisti” curd), and relative risk (RR) of resistance at the ripened stage.

Antibiotic	(n) of Resistant Strains 5-Day Curd ^a^	(n) of Resistant Strains 30-Day Curd	(RR) ^b^	CI ^c^ 95%
Ampicillin	190 (36.68%)	318 (55.99%)	1.82	1.62–2.10
Sulbactam/ Ampicillin	53 (10.23%)	91 (16.05%)	1.57	1.14–2.15
Erythromycin	125 (24.13%)	42 (7.41%)	0.31	0.22–0.42
Clindamycin	73 (14.09%)	84 (14.81%)	1.05	0.79–1.41
Oxytetracycline	112 (21.62%)	60 (10.58%)	0.49	0.37–0.65
Chloramphenicol	35 (6.76%)	220 (38.80%)	5.74	4.10–8.04
Gentamycin	78 (15.06%)	72 (12.69%)	0.84	0.63–1.14
Streptomycin	31 (5.98%)	74 (13.05%)	2.18	1.46–3.26
Vancomycin	48 (9.27%)	146 (25.74%)	2.78	2.05–3.76
Teicoplanin	35 (6.76%)	84 (14.81%)	2.19	1.51–3.19
Fusidic acid	69 (13.32%)	66 (11.64%)	0.87	0.64–1.19
Metronidazole	30 (5.79%)	252 (44.45%)	7.67	5.36–10.99
Quinupristin/ Dalfopristin	30 (5.79%)	135 (23.81%)	4.11	2.82–5.99
Trimethoprim	32 (6.18%)	85 (14.99%)	2.43	1.65–3.58
Total (*)	362	438		

Notes: ^a^: Values are given as n (%) resistant isolates for each fermentation stage. Percentages are calculated relative to the total isolates recovered from each stage (5-day curd: n = 518; 30-day curd: n = 567); (*): The total does not refer to the product of the addition of the resistant strains of this table because many of them are MDR and would be counted more than once. ^b^: RR was calculated as the proportion resistant at day 30 divided by the proportion resistant at day 5 (RR > 1 indicates increased resistance at day 30; RR < 1 indicates decreased resistance at day 30); ^c^: CI, 95% confidence interval for RR. Differences were considered statistically significant when the 95% CI did not include 1.

**Table 6:** MIC distributions and experimental cut-off values (mg/L) for *L. acidophilus* isolates recovered from the 5–7-day “Tyrovolia” curd and the ≥30-day “Kopanisti” curd.

	5th Day Curd				30th Day Curd			
Antibiotic	MIC Range	MIC_50_	MI_90_	Cut Off	MIC Range	MIC_50_	MIC_90_	Cut Off
Ampicillin	0.03–1	0.5	0.5	0.12	0.12–1	0.5	1	-
Sulbactam/Ampicillin	0.12–0.5	0.12	0.5	-	0.06–2	0.5	1	0.12
Erythromycin	0.03–0.25	0.06	0.25	0.12	0.03–0.5	0.12	0.5	-
Clindamycin	0.12–0.5	0.25	0.5	-	0.12–0.5	0.12	0.25	-
Oxytetracycline	0.12–1	0.5	1	0.25	0.25–16	2	8	-
Chloramphenicol	0.12–0.5	0.25	0.5	-	0.12–8	0.5	2	2
Gentamycin	0.12–0.5	0.25	0.5	0.25	0.25–2	1	2	-
Streptomycin	1–8	4	8	-	0.5–32	4	16	-
Vancomycin	0.12–0.5	0.5	0.5	-	0.25–1	1	1	0.5
Teicoplanin	128–256	256	256	-	16–128	64	128	-
Fusidic acid	16–64	32	64	-	16–64	2	16	-
Metronidazole	32–500	64	256	128	64–≥500	≥500	≥500	-
Trimethoprim	0.25–1	0.5	1	-	2–256	16	256	4
Quinupristin/ dalfopristin	0.12–0.5	0.12	0.5	-	0.06–0.5	0.12	0.5	-

Notes: MIC, minimum inhibitory concentration; MIC_50_, concentration inhibiting 50% of isolates; MIC_90_, concentration inhibiting 90% of isolates.

**Table 7:** Detected resistance genes and corresponding phenotypes in *L. acidophilus* isolates recovered from the 5–7-day and ≥30-day curd matrices.

Gene	5th Day Matrix		30th Day Matrix	
	n	Phenotype	n	Phenotype
*tet*M	9 (12.86%) *	9 resistant to oxytetracycline	5 (7.04%) *	5 susceptible to oxytetracycline
*tet*K	6 (8.57%)	5 resistant + 1 susceptible to oxytetracycline	0	-
*cat*	0	-	4 (5.63%)	2 resistant + 2 susceptible to chloramphenicol
*blaTEM*	3 (4.29%)	All 3: Ampicillin resistant Sulbactam/Ampicillin susceptible	7 (9.86%)	6 resistant to Ampicillin and Sulbactam/Ampicillin & 1 Ampicillin susceptible and Sulbactam/Ampicillin resistant
*erm*B	3 (4.29%)	3 resistant to erythromycin	3 (4.22%)	3 susceptible to erythromycin
Total	21	-	19	-

(*): Percentage of the total *L. acidophilus* isolates recovered from the corresponding matrix. Abbreviations: *bla*TEM, gene encoding a beta-lactamase; erm*B*, gene encoding a 23S rRNA methyltransferase; *tet*M, gene encoding a ribosomal protection protein; *tet*K, gene encoding a tetracycline efflux pump; *cat*, gene encoding chloramphenicol acetyltransferase.

**Table 8:** Combined analysis of phenotypic resistance and corresponding resistance genes in *Lactobacillus acidophilus* isolates (n = 141).

Determining Resistance to Antibiotic Phenotype	Resistance Gene	Phenotypically Resistant, n/N (%)	Gene-Positive Isolates, n/N (%)	Resistant and Gene-Positive, n/N (%)	Gene-Positive Among Resistant, n (%)	Fisher’s Exact *p*-Value
Ampicillin	*bla*TEM	104/141 (73.8)	10/141 (7.1)	9/141 (6.4)	9/104 (8.7)	0.455
Sulbactam/Ampicillin	*bla*TEM	43/141 (30.5)	10/141 (7.1)	6/141 (4.3)	6/43 (14.0)	0.067
Erythromycin	*erm*B	36/141 (25.5)	6/141 (4.3)	3/141 (2.1)	3/36 (8.3)	0.174
Oxytetracycline	*tet*M	36/141 (25.5)	14/141 (9.9)	9/141 (6.4)	9/36 (25.0)	0.001
Oxytetracycline	*tet*K	36/141 (25.5)	6/141 (4.3)	5/141 (3.5)	5/36 (13.9)	0.004
Chloramphenicol	*cat*	12/141 (8.5)	4/141 (2.8)	2/141 (1.4)	2/12 (16.7)	0.036

Abbreviations: *bla*TEM, beta-lactamase gene; *erm*B, 23S rRNA methyltransferase gene; *tet*M, ribosomal protection gene; *tet*K, tetracycline efflux pump gene; *cat*, chloramphenicol acetyltransferase gene. Footnotes: Gene-positive indicates PCR detection of the corresponding resistance determinant. Phenotypically resistant indicates isolates scored as resistant in the binary dataset. Associations between phenotype and genotype were evaluated using Fisher’s exact test. *p*-values < 0.05 were considered statistically significant. For oxytetracycline resistance, both *tet*M and *tet*K were evaluated separately because they represent distinct resistance mechanisms.

## Data Availability

The original contributions presented in this study are included in the article/Supplementary Material. Further inquiries can be directed to the corresponding authors.

## Supplementary Materials

The following supporting information can be downloaded at: https://www.mdpi.com/article/10.3390/microorganisms14030712/s1, Supplementary Material; Table S1: Primer sequences and PCR assay conditions for screening antibiotic resistance genes; Table S2: MIC values range and experimental cut off values of the Lactobacilli strains isolated from the 5th day curd; Table S3: MIC values range and experimental cut off values of the Lactobacilli strains isolated from the 30th day curd; Table S4: Alterations in the upper limit of the MIC range from the 5th to the 30th day; Table S5: Proposed cut-off MIC values by EFSA (2018) for *Lactobacillus* spp. [31]; Table S6: Resistant strains (n) by EFSA breakpoints and by the experimental cut off values of this study; Table S7: Lactobacilli taxa isolated in this study and their reported occurrence across potential One Health reservoirs (livestock, humans, companion animals, and environmental niches) based on the published literature. Refs. [53,54,55,56,57,58,59,60,61,62,63,64,65,66,67,68,69,70,71,72,73,74,75,76,77,78,79,80,81,82,83,84,85,86,87,88,89,90,91,92,93,94,95,96,97,98,99,100,101,102,103,104,105,106,107,108,109,110,111,112] are cited in Supplementary Materials.
