# Supplementary material for: One Health Investigation of Stage-Dependent Antimicrobial Resistance Patterns Across Intermediate and Ripened Dairy Matrices: The Tyrovolia–Kopanisti Paradigm
Source: Microorganisms. 2026 Mar 22;14(3):712. doi: 10.3390/microorganisms14030712 (PMC13028824; doi:10.3390/microorganisms14030712)
Supplement: Supplementary file 1 [file microorganisms-14-00712-s001.zip › S1.pdf]

Table S1: Primer sequences and PCR assay conditions for screening antibiotic resistance genes

a/a	Gene targeted	Primer sequence (5'→3')	Amplicon length (bp)	Annealing temperature (◦C)	References
1	*tet*M	F: GCTTGATCCCCAGTAAGTCA	401	55	23,24
		R: GGTGAACATCATAGACACGC			
2	*tet*K	F: TTATGGTGGTTGTAGCTAGAAA	348	55	25,26
		R: AAAGGGTTAGAAACTCTTGAAA			
3	*erm*B	F: CATTTAACGACGAAACTGGC	425	60	27
		R: GGAACATCTGTGGTATGGCG			
4	*bla*TEM	F: ATCAGCAATAAACCAGC	516	54	28,26
		R: CCCCGAAGAACGTTTTC			
5	*cat*	F: TTAGGTTATTGGGATAAGTTA	300	52	29,30
		R: GCATGRTAACCATCACAWAC			
