# Supplementary material for: One Health Investigation of Stage-Dependent Antimicrobial Resistance Patterns Across Intermediate and Ripened Dairy Matrices: The Tyrovolia–Kopanisti Paradigm
Source: Microorganisms. 2026 Mar 22;14(3):712. doi: 10.3390/microorganisms14030712 (PMC13028824; doi:10.3390/microorganisms14030712)
Supplement: Supplementary file 1 [file microorganisms-14-00712-s001.zip › S2.pdf]

Table S2: MIC values range and experimental cut off values of the *Lactobacilli* strains isolated from the 5th day curd

***Lactobacillus* spp**	**Antibiotics (mg/L)**													
	**Amp**	**Sulb/ Amp**	**Ery**	**Clin**	**Oxy**	**Clor**	**Gen**	**Str**	**Van**	**Tei**	**Fus**	**Metro**	**Tri**	**Q/D**
L. helveticus	0.03-0.06	0.08-0.12	0.06	0.25	0.5-2	0.5-2	0.25-0.5	4-16	0.12-0.25	0.06-0.12	2-8	128-256	0.25-1	0.03-0.12
L. acidophilus	0.03-1 (0.12)	0.12-0.5	0.03-0.25 (0.12)	0.12-0.5	0.12-1 (0.25)	0.12-0.5	0.12-0.5 (0.25)	1-8	0.12-0.5	128-256	16-64	32-≥500 (128)	0.25-1	0.12-0.5
L. paraplantarum	0.25-1 (0.5)	0.5-2 (1)	0.03-0.5 (0.06)	0.03-0.25	2-8	0.5-4 (2)	0.5-2	2-16	16-32	8-16	1-8 (4)	128-256	0.25-16 (2)	0.12-0.5
L. brevis	0.5-1	1	1-2	0.03	0.25-0.5	1-2	0.25-0.5	4-32 (8)	0.25-0.5	2-4	0.5-16 (1)	64-128	4-16 (8)	0.06-0.12
L. delbrueckii subsp *bulgaricus*	0.06-0.5 (0.12)	0.12-4 (0.5)	0.03-0.25 (0.06)	0.06-0.25	4-16 (8)	1-4	1-2	2-16	0.25-0.5	0.06-0.25 (0.12)	16-64	128-256	64-128	0.06-0.5 (0.25)
L. johnsonii	0.03-0.06	0.03-0.06	0.03	0.06-0.12	4	1-2	0.25-1	1-4	0.25-0.5	0.03-0.06	64-256	128-256	4-8	0.06-0.25
L. curvatus	0.12-0.5 (0.25)	0.06-0.25	0.06-0.12	0.06-0.12	0.25-1	0.25-0.5	0.25-0.5	16-64	0.12-1 (0.25)	0.06-0.12	0.25-1	128-≥500 (256)	4-8	0.12-0.25
L. salivarius	0.03-0.06	0.03-0.06	0.03	0.06-0.12	0.5-1	0.25-0.5	0.06-0.12	0.5-1	0.12-0.25	0.12-1 (0.25)	0.12-0.25	32-64	2-4	0.03-0.06
L. plantarum	0.12-0.5	0.5-1	0.06-0.25	0.12-0.25	2-8 (4)	1-2	0.5-2 (1)	4-16	16-32	16-32	1-2	128-256	0.5-1	0.12-0.25
L. rhamnosus	0.12-0.5	0.06-0.12	0.03	0.03-0.06	0.25-1	1-4	0.25-0.5	1-4	8-16	2-4	32-128	32-64	16-64	0.12-0.25
L. delbrueckii subsp *lactis*	0.06-0.5	0.06-0.25 (0.12)	0.12-0.25	0.03-0.25	0.25-2 (1)	1-2	0.12-1 (0.5)	1-8	0.12-0.25	0.06-0.25	4-64 (16)	64-256	32-64	0.06-0.25
L. pentosus	0.03-2 (0.12)	0.06-4 (0.25)	0.06-0.5 (0.12)	0.03-0.12 (0.06)	2-8 (4)	0.25-2	0.25-1	4-64 (16)	0.25-32 (0.5)	2-16 (4)	2-16	128-256	0.5-16 (4)	0.03-0.25
L. casei subsp *casei*	0.25-0.5	0.5-1	0.25-0.5	0.06	1-2	2	1-2	2-4	8-16	8-16	32-64	128-256	8-16	0.25-0.5
L. casei subsp *pseudoplantarum*	2	0.5-1	0.06-0.12	0.03-0.06	1	0.5-1	1	2-4	4	16	8-16	256	4-8	0.5
L. sakei	0.03-0.12	0.03-0.06	0.03-0.12 (0.06)	0.03-0.12 (0.06)	0.5-1	0.25-1 (0.5)	0.12-0.25	1-2	0.06-0.5	0.5-1	0.12-1	64-256	0.12-0.25	0.03-0.06
L. fermentum	0.06-1 (0.25)	0.06-1 (0.25)	0.03-0.06	0.03-0.06	0.5-2	0.5-1	0.25-0.5	0.5-4 (1)	4-32 (16)	2-8	0.06-0.5 (0.12)	64-256	16-64	0.06-0.25

(*): in parenthesis the experimental cut off value
