# Supplementary material for: One Health Investigation of Stage-Dependent Antimicrobial Resistance Patterns Across Intermediate and Ripened Dairy Matrices: The Tyrovolia–Kopanisti Paradigm
Source: Microorganisms. 2026 Mar 22;14(3):712. doi: 10.3390/microorganisms14030712 (PMC13028824; doi:10.3390/microorganisms14030712)
Supplement: Supplementary file 1 [file microorganisms-14-00712-s001.zip › S3.pdf]

Table S3: MIC values range and experimental cut off values of the *Lactobacilli* strains isolated from the 30th day curd

***Lactobacillus* spp**	**Antibiotics (mg/L)**													
	**Amp**	**Sulb/ Amp**	**Ery**	**Clin**	**Oxy**	**Clor**	**Gen**	**Str**	**Van**	**Tei**	**Fus**	**Met**	**Tri**	**Q/D**
L. helveticus	0.12-0.5	0.12-1	0.12-0.5	0.06-0.25	0.25-2 (1)	0.12-4 (0.25)	0.25-2	4-32	0.12-0.5	0.25-0.5	8-32	128-≥500	2-32	0.03-0.5 (0.12)
L. acidophilus	0.12-1	0.06-2 (0.12)	0.03-0.5	0.12-0.5	0.25-16	0.12-8 (2)	0.25-2	0.5-32	0.25-1 (0.5)	16-128	16-64	64-≥500	2-256 (4)	0.06-0.5
L. paraplantarum	0.25-2	0.12-0.5	0.06-0.5 (0.12)	0.03-0.25 (0.06)	8-16	0.25-4 (1)	0.25-2	1-8 (2)	8-64 (16)	4-32 (8)	1-16 (4)	128-≥500	0.5-32	0.12-0.5
L. brevis	0.12-0.5	0.06-1 (0.5)	0.12-1	0.03-0.06	0.5-32 (2)	0.25-2	0.25-0.5	1-8	0.5-2	2-16	0.25-0.5	32-128	4-8	0.03-0.25
L. delbrueckii subsp *bulgaricus*	0.06-2 (0.5)	0.12-2	0.03-0.5	0.06-0.5	2-16	1-16 (4)	0.5-8 (2)	4-32	0.12-2 (0.25)	0.06-0.25	16-128	128-≥500	32-256	0.03-0.5 (0.06)
L. johnsonii	0.06-0.5	0.12-0.5	0.03-0.06	0.06-0.25	2-8	0.5-8 (2)	0.5-2	1-8 (2)	0.25-0.5	0.06-0.12	84-256	128-≥500	8-128	0.06-0.25
L. curvatus	0.12-2 (0.5)	0.12-0.5	0.03-0.25	0.03-0.12	0.25-8 (0.5)	0.5-4	0.5-2	32-128	8-64 (32)	0.06-0.25	32-128	256-≥500	32-64	0.03-8 (1)
L. salivarius	0.12-0.25	0.06-0.25 (0.12)	0.06-0.12	0.06-0.5 (0.12)	0.12- 0.5	0.25-1	0.12-0.5	0.25-1	0.25-0.5	0.25	0.12-4 (0.25)	64-256 (128)	4-16	0.03
L. plantarum	0.25-4	0.06-1	0.06-0.5	0.03-0.5 (0.06)	2-16	1-4	0.5-2	8-32	16-128	8-256 (16)	1-16 (4)	256-≥500	8-16	0.06-1 (0.25)
L. rhamnosus	0.12-0.5	0.12-0.5 (0.25)	0.03-0.12	0.03-0.12	0.06-0.25	0.5-4 (1)	0.25-2 (0.5)	4-64	128-≥500 (≥500)	8-64	16-64	32-256 (128)	32-128	0.06-0.12
L. delbrueckii subsp *lactis*	0.03-0.12	0.06-0.25	0.03-0.12	0.03-0.12	0.12-1 (0.5)	1-2	0.25-16 (4)	4-8	0.25-1	0.12-0.25	8-32	64-256 (128)	16-128	0.06-0.25
L. pentosus	0.5-1	1	0.06	0.03-0.06	16-64	1-4 (2)	1-4	32-64	64-128	32-64	0.06-0.25	256	1-4 (2)	0.06-0.5 (0.12)
L. casei subsp *casei*	0.12-0.25	0.5-1	0.06	0.06-0.12	2-4	2-8	1-4	2-8	64-128	18-32	16-32	64-256 (128)	4-8	0.12-0.5
L. casei subsp *pseudoplantarum*	0.25-0.5	0.5-1	0.25-0.5	0.03-0.06	4-8	2-4	2	2-4	128-256	64-128	8-16	128-256	2-4	0.5-1
L. sakei	0.03-0.25 (0.12)	0.03-0.06	0.03-0.12	0.06-0.12	0.25-0.5	0.12-1 (0.25)	0.06-0.25	0.5-1	0.25-2	0.12-0.5	0.12-1 (0.25)	64-256	0.5-2 (1)	0.03-0.06
L. fermentum	0.06-0.12	0.06-0.25	0.06-0.12	0.03-0.06	0.25-2 (1)	0.5-1	0.5-1	1-8 (2)	32-128	4-8	0.06-0.25	128-256	16-32	0.06-0.5

(*): in parenthesis the experimental cut off values
