# Supplementary material for: One Health Investigation of Stage-Dependent Antimicrobial Resistance Patterns Across Intermediate and Ripened Dairy Matrices: The Tyrovolia–Kopanisti Paradigm
Source: Microorganisms. 2026 Mar 22;14(3):712. doi: 10.3390/microorganisms14030712 (PMC13028824; doi:10.3390/microorganisms14030712)
Supplement: Supplementary file 1 [file microorganisms-14-00712-s001.zip › S4.pdf]

Table S4: Alterations in the upper limit of the MIC range from the 5th to the 30th day

Lactobacillus spp	**Antibiotics**														
	**Pen G**	**Amp**	**Sulb / Amp**	**Ery**	**Clin**	**Oxy**	**Clor**	**Gen**	**Str**	**Van**	**Tei**	**Fus**	**Met**	**Tri**	**Q/D**
L. helveticus	I*	I	I	I	=	=	I	I	I	I	I	I	I	I	I
L. acidophilus	I	=	I	I	=	I	I	I	I	I	I	=	=	I	=
L. paraplantarum	D	I	D	=	=	I	=	=	D	I	I	I	I	I	=
L. brevis	I	D	=	D	I	I	=	=	D	I	I	D	=	D	I
L. delbrueckii subsp *bulgaricus*	D	I	D	I	I	=	I	I	I	I	=	I	I	I	=
L. johnsonii	I	I	I	I	I	I	I	I	I	=	I	=	I	I	=
L. curvatus	I	I	I	I	=	I	I	I	I	I	I	I	=	I	I
L. salivarius	I	I	I	I	I	D	I	I	=	I	D	I	I	I	D
L. plantarum	I	I	=	I	I	I	I	=	I	I	I	I	I	I	I
L. rhamnosus	I	=	I	I	I	D	=	I	I	I	I	D	I	I	D
L. delbrueckii subsp *lactis*	D	D	=	D	D	D	=	I	=	I	=	D	=	I	=
L. pentosus	D	D	D	D	D	I	I	I	=	I	I	D	=	D	I
L. casei subsp *casei*	D	D	=	D	I	I	I	I	I	I	I	D	=	D	=
L. casei subsp *pseudoplantarum*	D	D	=	I	=	I	I	I	=	I	I	=	=	D	I
L. sakei	I	I	=	=	=	D	=	=	D	I	I	=	=	I	=
L. fermentum	D	D	D	I	=	=	=	I	I	I	=	D	=	D	I

(*): I for increase of the upper limit; D: for decrease of the upper limit; and (=): for equality
