# Supplementary material for: One Health Investigation of Stage-Dependent Antimicrobial Resistance Patterns Across Intermediate and Ripened Dairy Matrices: The Tyrovolia–Kopanisti Paradigm
Source: Microorganisms. 2026 Mar 22;14(3):712. doi: 10.3390/microorganisms14030712 (PMC13028824; doi:10.3390/microorganisms14030712)
Supplement: Supplementary file 1 [file microorganisms-14-00712-s001.zip › S5.pdf]

Table S5: Proposed cut-off MIC values by EFSA (2018) for *Lactobacillus* spp [31]

**Lactobacillus groups and species**	**Antibiotics**							
	Amp	Van	Gen	Str	Ery	Clin	Tet	Chlor
Obligate homofermentive<br>*L. helveticus,*<br>*L. delbrueckii* subsp *bulgaricus,*<br>*L. delbrueckii* subsp *lactis*	2	2	16	16	1	4	4	4
L. acidophilus group (includes *L. johnsonii*)	1	2	16	16	1	4	4	4
Obligate heterofermentive<br>*L. pentosus*<br>*L. fermentum*<br>*l. brevis*	2	n. r.	16	64	1	4	8	4
Facultative heterofermentive<br>*L. curvatus*<br>*L. paraplantarum*<br>*L. sakei*	4	n. r.	16	64	1	4	8	4
L. plantarum<br>*L. pentosus*	2	n. r.	16	n. r.	1	4	32	8
L. rhamnosus	4	n. r.	16	n. r.	1	4	8	4
L. casei subsp *casei*<br>*L. casei* subsp *pseudoplantarum*	4	n. r.	32	64	1	4	4	4
