# Supplementary material for: One Health Investigation of Stage-Dependent Antimicrobial Resistance Patterns Across Intermediate and Ripened Dairy Matrices: The Tyrovolia–Kopanisti Paradigm
Source: Microorganisms. 2026 Mar 22;14(3):712. doi: 10.3390/microorganisms14030712 (PMC13028824; doi:10.3390/microorganisms14030712)
Supplement: Supplementary file 1 [file microorganisms-14-00712-s001.zip › S6.pdf]

Table S6: Resistant strains (n) by EFSA breakpoints and by the experimental cut off values of this study

**Antibiotic**	**5th day matrix**		**30th day matrix**	
	**EFSA breakpoint**	**Experimental cut off**	**EFSA breakpoint**	**Experimental cut off**
**Ampicillin**	0	166	9	62
**Erythromycin**	8	125	0	42
**Clindamycin**	0	73	0	84
**Oxytetracycline**	34	112	107	60
**Chloramphenicol**	0	35	86	220
**Gentamicin**	0	78	6	72
**Streptomycin**	1	31	40	74
**Vancomycin**	0	48	0	146
