# Supplementary material for: One Health Investigation of Stage-Dependent Antimicrobial Resistance Patterns Across Intermediate and Ripened Dairy Matrices: The Tyrovolia–Kopanisti Paradigm
Source: Microorganisms. 2026 Mar 22;14(3):712. doi: 10.3390/microorganisms14030712 (PMC13028824; doi:10.3390/microorganisms14030712)
Supplement: Supplementary file 1 [file microorganisms-14-00712-s001.zip › S7.pdf]

Table S7: Lactobacilli taxa isolated in this study and their reported occurrence across potential One Health reservoirs (livestock, humans, companion animals, and environmental niches) based on the published literature

**Site**	**Species**	**Table-supporting references**
Livestock GI tract	*L. acidophilus, L. salivarius, L. plantarum, L. fermentum, L. johnsonii. L. pentosus, L. delbrueckii* subsp *lactis. L. curvatus, L. casei* subsp *pseudoplantarum*	[53-62]
Livestock oral cavity	*L. acidophilus, L. fermentum, L. plantarum, L. salivarius, L. rhamnosus*	[63-65]
Livestock udder	*L. casei, L. plantarum, L. fermentum, L. rhamnosus, L. brevis*	[66-69]
Livestock vagina	*L. plantarum, L. fermentum*	[70,71]
Livestock skin	*L. plantarum, L. fermentum, L. rhamnosus, L. casei, L. brevis*	[72-74]
Livestock nasal cavity	*L. casei, L. rhamnosus, L. plantarum*	63
Human skin	*L. plantarum, L. fermentum, L. rhamnosus, L. casei*	[75,76]
Human oral cavity	*L. fermentum, L. rhamnosus, L. casei, L. salivarius, L. plantarum, L delbrueckii* subsp *bulgaricus*	[77-82]
Human GI tract	*L. acidophilus, L. casei, L. plantarum, L. rhamnosus, L. salivarius, L. fermentum*	[59,83,84]
Human nasal cavity	*L. rhamnosus, L. casei, L. sakei, L. plantarum*	[85-87]
Canine skin	*L. johnsonii, L. fermentum, L. plantarum, L. brevis*	[88-80]
Canine oral cavity	*L. fermentum, L. plantarum, L. casei, L. salivarius, L. rhamnosus, L. acidophilus, L. delbrueckii* subsp *bulgaricus*	[91,92]
Canine GI tract	*L. acidophilus, L. rhamnosus, L. plantarum, L. fermentum, L. salivarius*	[90-95]
Feline GI tract	*L. acidophilus, L. rhamnosus, L. johnsonii, L. casei, L. plantarum*	[59, 95-97]
Feline oral cavity	*L. acidophilus, L. casei, L. plantarum, l. rhamnosus*	[59,98]
Plants, vegetation, rhizosphere	*L. plantarum, L. brevis, L. fermentum, L. paraplantarum, L. pentosus, L. curvatus, L. casei* subsp *pseudoplantarum*	[99-106]
Soil	*L. plantarum, L. brevis, L. casei, L. fermentum, l. rhamnosus*	[107-112]
